# Left-Sided Congenital Diaphragmatic Hernia with Multiple Congenital Cardiac Anomalies, Hernia Sac, and Microscopic Hepatic Heterotopia: A Case Report

**DOI:** 10.4061/2011/967107

**Published:** 2011-03-15

**Authors:** Maria Arafah, Deena T. Boqari, Khaled O. Alsaad

**Affiliations:** ^1^Department of Pathology, King Khalid University Hospital, College of Medicine, King Saud University, Saudi Arabia; ^2^Department of Pathology and Laboratory Medicine, King Abdulaziz Medical City, Saudi Arabia; ^3^The College of Medicine, King Saud bin Abdulaziz University for Health Sciences and King Abdullah International Research Centre, P.O. Box 22490, Riyadh 11426, Saudi Arabia

## Abstract

Congenital diaphragmatic hernia is a common congenital anomaly of uncertain etiology. Its association with multiple congenital anomalies in various organs is well recognized and antenatal radiological evidence of congenital diaphragmatic hernia warrants thorough evaluation to detect other anomalies, some of which can be life threatening. Rarely, heterotopic hepatic tissue is identified in the hernia, a rare pathological finding, exhibiting more than one macroscopic and microscopic characteristics, and always associated with cardiac congenital anomalies. Herein, we report a case of left-sided microscopic heterotopic hepatic tissue in a congenital diaphragmatic hernia in an infant with multiple cardiac congenital anomalies, but with preserved pericardium.

## 1. Introduction

Congenital diaphragmatic hernia (CDH) is a common, life-threatening congenital anomaly with an incidence ranging between 1 in 2000 and 1 in 5000 live birth [1]. The pathogenesis of CDH remains unclear, and its association with various concurrent congenital anomalies and variable pathological patterns and clinical presentation suggests that CDH is a result of multiple, complex developmental abnormalities [2]. It is estimated that associated congenital anomalies present in 30 to 40% of the cases of CDH [3], of which the most common are those of cardiac, urinary tract, gastrointestinal tract and central nervous system defects, as well as skeletal and neural tube defects [4, 5]. Heterotopic liver tissue in CDH is rare and can be present in different macroscopic and microscopic forms. Microscopic heterotopic hepatic tissue in CDH is a very uncommon finding and almost always associated with cardiac congenital anomalies, particularly absence of the pericardium [6, 7]. We report a case of a left-sided CDH with a microscopic heterotopic hepatic tissue and multiple cardiac congenital malformations but with a preserved pericardium. 

## 2. Case Report

**Figure 1:**
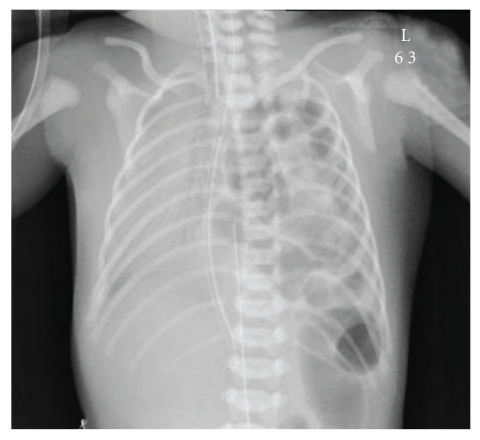
Chest X-ray shows left-sided congenital diaphragmatic hernia with herniating loops of large bowel into the right hemithorax.

 A full-term infant boy was delivered spontaneously and vaginally after an uneventful pregnancy. Antenatal ultrasound revealed left CDH. His Apgar score was 6 and 7 at 1 and 5 minutes, respectively. He immediately developed respiratory distress and was intubated and transferred to the neonatal intensive care unit. Chest X-ray confirmed the left-sided CDH and showed pouching out of the loops of the large bowel, mediastinal shift to the right, and right lung hypoplasia (Figure 1). Initial echocardiogram revealed a small patent ductus arteriosus with no other cardiac congenital anomalies. At the age of 14 days, the infant underwent a surgical repair of the CDH, of which the hernia was reduced back into the abdomen, and the hernia sac was excised. Intraoperatively, both lobes of the liver were intraabdominal. 

**Figure 2:**
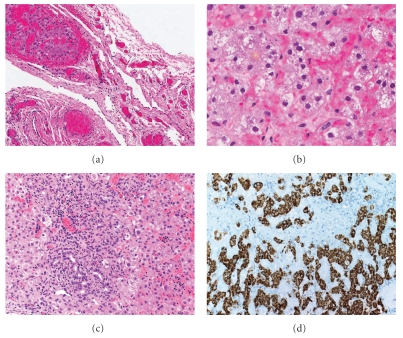
(a) Vascularized hernia sac with foci of heterotopic hepatic tissue. (b) Congested sinusoidal spaces separating the hepatic plates. (c) Areas of cholangiolar proliferation with accompanying mixed inflammatory cell infiltrate were seen. Note the mild hydropic change in the hepatocytes and the extramedullary hematopoiesis. (d) Immunexpression of Hep Par-1 in the heterotopic hepatocytes (original magnification (a) X100; (b) X400; (c) X200, (d) X200).

Grossly, the specimen consisted of a smooth, thick, and transparent sac-like structure measuring 4.5 cm in the maximum dimension. Multiple small tan-brown dark foci were noted in the sac-like structures, but no solid areas or papillary projections were obvious. Histological examination of formalin-fixed, paraffin embedded 5 *μ*m, hematoxylin and eosin-stained sections showed vascularized fibrous tissue lined by bland mesothelial cells. Sections from the dark foci showed microscopic heterotopic hepatic tissue (Figure 2(a)). The hepatocytes were arranged in thin plates and focally in solid sheets. Congested sinusoidal spaces lined by flat Kupffer cells were seen separating the hepatic plates (Figure 2(b)). The hepatocytes showed a moderate degree of hydropic degeneration (ballooning), and focal canalicular cholestasis was noted. Areas recapitulating portal tracts were focally noted in the heterotopic hepatic tissue, but lacked intrahepatic arterioles. Areas in which florid cholangiolar proliferation, associated with mixed inflammatory cell infiltrate were identified (Figure 2(c)). The inflammatory cells, were predominantly lymphocytes and neutrophils; scattered eosinophils were also noted. Extramedullary hematopoiesis was noted. Immunohistochemically, the hepatocytes were strongly reactive for Hep Par-1 antibody (clone OCH1E5, dilution 1 : 50, Dako, Denmark) (Figure 2(d)). No heterotopic tissue other than hepatic tissue was identified. 

The postoperative clinical course was complicated by Pseudomonas aeruginosa pneumonia. The patient was intubated and readmitted to the neonatal intensive care unit, where he was successfully treated with antibiotics. Further clinical and radiological work up revealed a patent foramen ovale and ventricular septal defect. The pericardium was unremarkable.

## 3. Discussion

The diaphragm is formed by the fusion of several embryonic components which include the septum transversum, pleuroperitoneal membranes, esophageal mesentery, and body wall mesoderm. As proposed by the work of Kluth et al. [8, 9], the development of the diaphragm is divided into two phases (1) the development of the diaphragmatic pericardium and (2) the development of the pleural cavity and the closure of the pleuroperitoneal canal (PPC). In the normal embryogenesis, the septum transversum separates the pericardial and the peritoneal cavities as it fuses dorsally with the mesodermal tissue surrounding the foregut. As this occurs, the PPC remains patent, connecting the pleural spaces and peritoneal cavity [10]. The pathogenesis of CDH is still not fully understood, and until recently, the general hypothesis is that the defect in CDH results from failure of complete closure of the PPC at the embryonic period 8th–10th gestational week, which is an essential embryological step that completes the formation of the primitive fetal diaphragm [9].

The liver primordium appears in the 4th gestational week, as an outgrowth of the endodermal epithelium at the distal end of the foregut, known as hepatic diverticulum, which consists of proliferating cell strands that penetrate the septum transversum. Iritani [11] indicated that a posthepatic mesenchymal plate (PHMP), which is the mesenchymal tissue that appears dorsal to the liver and ventral to the peritoneal canal, plays a cardinal role in the development of the diaphragm, and its growth is responsible for the closure of the PPC. The work of Kluth et al. [8, 9] using a nitrofen-induced rat model to study the cellular mechanisms of CDH showed failure of normal growth of the PHMP. Recent advances in cytogenetic analysis revealed various chromosomal aberrations and their association with CDH, and minimally deleted region for CDH has been identified on chromosome 15q26.1-26.2 [12]. *COUP-TFII*, a member of nuclear receptors, is one of the genes located within this region. COUP-TFII is expressed in the primitive foregut mesenchyme, the developing PHMP, developing lung, and the septum transversum, the components that are important for the formation of the diaphragm. As elegantly demonstrated by the work of You et al. [13], ablation of* COUP-TFII* leads to abnormal development of PHMP and might play a critical role in the development of diaphragmatic hernia in mice. Therefore, it is likely that *COUP-TFII* contributes to the formation of CDH in individuals with 15q deletions. 

Heterotopic liver tissue was reported in various organs, such as gallbladder, adrenal gland, small intestine, heart and lung as well as pleura and pericardium [14–19]. Collan at al [20] classified abnormally positioned liver tissue into four types: (1) accessory liver lobe that can reach a considerable size and is attached to the liver by a stalk, (2) small accessory liver lobe, which is attached to the liver but is usually small (10–30 grams in weight), (3) ectopic liver without any connection with it, and (4) microscopic ectopic liver. Types 2 and 3 have been reported to occur in association with CDH [21, 22]. 

Microscopic heterotopic liver tissue in a sac of a diaphragmatic hernia was rarely reported. To the best of our knowledge, only two cases are described in the English literature [6, 7], both of which were associated with congenital absence of the pericardium, along with other congenital cardiac anomalies. The current case, as well as the previous two cases, is characterized by the presence of thick hernia sac containing microscopic foci of hepatic tissue. The hepatic tissue is considered heterotopic because it is histologically normal tissue found in an abnormal anatomical location. A recent report described a case of CDH in an infant, of which the left attenuated side of the liver formed a part of the dome of the hernia sac, and the wall of the hernia sac contained cord-like structures histologically confirmed to be liver tissue [23]. The association between CDH and congenital cardiac anomalies, including absent pericardium, is well recognized. Despite the presence of patent foramen ovale and ventricular septal defect in the current case, the pericardium was intact, a finding that has not been reported before in association with the presence of microscopic heterotopic liver tissue in a CDH. This emphasizes the diversity of possible mechanisms of CDH as well as its clinical consequences and outcome.
